# Perinatal Na^+^ Overload Programs Raised Renal Proximal Na^+^ Transport and Enalapril-Sensitive Alterations of Ang II Signaling Pathways during Adulthood

**DOI:** 10.1371/journal.pone.0043791

**Published:** 2012-08-22

**Authors:** Edjair V. Cabral, Leucio D. Vieira-Filho, Paulo A. Silva, Williams S. Nascimento, Regina S. Aires, Fabiana S. T. Oliveira, Ricardo Luzardo, Adalberto Vieyra, Ana D. O. Paixão

**Affiliations:** 1 Department of Physiology and Pharmacology, Federal University of Pernambuco, Recife, Brazil; 2 Carlos Chagas Filho Biophysics Institute, Federal University of Rio de Janeiro, Brazil; 3 National Institute of Science and Technology for Structural Biology and Bioimaging, Rio de Janeiro, Brazil; The University of Manchester, United Kingdom

## Abstract

**Background:**

High Na^+^ intake is a reality in nowadays and is frequently accompanied by renal and cardiovascular alterations. In this study, renal mechanisms underlying perinatal Na^+^ overload-programmed alterations in Na^+^ transporters and the renin/angiotensin system (RAS) were investigated, together with effects of short-term treatment with enalapril in terms of reprogramming molecular alterations in kidney.

**Methodology/Principal Findings:**

Male adult Wistar rats were obtained from dams maintained throughout pregnancy and lactation on a standard diet and drinking water (control) or 0.17 M NaCl (saline group). Enalapril (100 mg/l), an angiotensin converting enzyme inhibitor, was administered for three weeks after weaning. Ninety day old offspring from dams that drank saline presented with proximal tubules exhibiting increased (Na^+^+K^+^)ATPase expression and activity. Ouabain-insensitive Na^+^-ATPase activity remained unchanged but its response to angiotensin II (Ang II) was lost. PKC, PKA, renal thiobarbituric acid reactive substances (TBARS), macrophage infiltration and collagen deposition markedly increased, and AT_2_ receptor expression decreased while AT_1_ expression was unaltered. Early treatment with enalapril reduced expression and activity of (Na^+^+K^+^)ATPase, partially recovered the response of Na^+^-ATPase to Ang II, and reduced PKC and PKA activities independently of whether offspring were exposed to high perinatal Na^+^ or not. In addition, treatment with enalapril *per se* reduced AT_2_ receptor expression, and increased TBARS, macrophage infiltration and collagen deposition. The perinatally Na^+^-overloaded offspring presented high numbers of Ang II-positive cortical cells, and significantly lower circulating Ang I, indicating that programming/reprogramming impacted systemic and local RAS.

**Conclusions/Significance:**

Maternal Na^+^ overload programmed alterations in renal Na^+^ transporters and in its regulation, as well as severe structural lesions in adult offspring. Enalapril was beneficial predominantly through its influence on Na^+^ pumping activities in adult offspring. However, side effects including down-regulation of PKA, PKC and AT_2_ receptors and increased TBARS could impair renal function in later life.

## Introduction

High Na^+^ intake is a reality of modern society, particularly due to the use of industrialized products. Rats subjected to maternal Na^+^ overload during prenatal and lactation periods present with glomerulosclerosis [1], increased proteinuria [2] and hypertension [3], [4] as adults. When exposed to Na^+^ overload during the prenatal period, newborn rats present with reduced expression of several markers of fetal kidney development including angiotensin II (Ang II) [5]. When exposed to Na^+^ overload from conception to weaning, the renin activity of adult offspring is unresponsive to a high salt intake, *i.e.* high Na^+^ intake does not suppress renin secretion and Ang II expression is increased in kidneys. Therefore, perinatal Na^+^ overload leads to renin angiotensin system (RAS) over-activity during adulthood [4]. In addition, an overactive RAS appears to be responsible, at least in part, for the aforementioned renal functional alterations produced by perinatal over-exposure to salt. Furthermore, Ang II increases renal oxidative stress [6], [7] that may disturb tubule interstitial microenvironment, leading to structural and functional changes in Na^+^ transporters [8], [9].

On the other hand, kidney development in the rat ends at approximately postnatal day 12 [10], and pharmacological inhibition of RAS during this period causes severe alterations in renal structure and function [11], [12], [13]. In humans, pharmacological inhibition of RAS during the second and third trimesters of pregnancy causes renal anomalies in offspring [14], [15], [16]. However, evidence suggests that short-term inhibition of RAS after weaning in rats could reverse prenatal programmed hypertension induced by maternal undernutrition [17]. In addition, it has been demonstrated that early maternal postnatal treatment with α-tocopherol prevents alterations in proximal tubule Na^+^ transporters of rats that were subjected to prenatal undernutrition [18]. Beneficial effects of inhibiting RAS after weaning demonstrates that the window of opportunity for imprinting molecular changes that affect renal function in adult life lasts beyond the conclusion of nephrogenesis and weaning [17], [18]. Therefore, various related early pathological processes can be reprogrammed to achieve normal profiles during adult life.

ATP-dependent Na^+^ transporters in the proximal tubule cells are modulated by RAS [19], [20]. Perinatal Na^+^ overload leads to RAS overactivity [4] that promotes increased oxidative stress [6] and may affect the activity of the proximal tubule ATP-dependent Na^+^ transporters. The present study was designed to determine whether a moderate perinatal Na^+^ overload produce late elevated tissular lipid peroxidation and local macrophage infiltration in the kidneys of young adulthood. The hypothesis was that these alterations could be associated with, or provoke, molecular alterations in: (i) the proximal tubule (Na^+^+K^+^)ATPase and Na^+^-ATPase; (ii) signaling pathways that link renal Ang II receptors, protein kinases C (PKC) and A (PKA), and active Na^+^ transporters. Furthermore, this study investigated whether inhibition of RAS for three weeks after weaning could reprogram perinatal programmed alterations in Na^+^ pumps, Ang II receptors (AT_1_ and AT_2_) expression, and the activity of PKC and PKA.

## Materials and Methods

### Animal care

Male Wistar rats were used throughout the study. Animal experimental procedures were approved by the Committee for Ethics in Animal Experimentation of the Federal University of Pernambuco, and carried out in accordance with Committee guidelines (protocol n° 23076.055063/2010-03).

### Materials

Enalapril maleate, thiobarbituric acid, furosemide, ouabain, Ang II, phenylmethanesulfonyl fluoride (PMSF), protein A-agarose and trypsin inhibitor (type II-S) were purchased from Sigma-Aldrich (St. Louis, MO). Calphostin C and PKA inhibitor (PKAi_5–24_) were obtained from Calbiochem (La Jolla, CA). Rabbit and goat polyclonal antibodies against Ang II receptors (AT_1_ and AT_2_) and the α-subunit of (Na^+^+K^+^)ATPase were purchased from Santa Cruz Biotechnology (Santa Cruz, CA), and that against the cellular marker for activated macrophages ED1 was obtained from AbD Serotec (Raleigh, NC). The polyclonal rabbit anti-Ang II was purchased from Peninsula Laboratories (San Carlos, CA). Streptavidin-horseradish peroxidase was obtained from Dako LSAB System (Dako Corporation, Carpinteria, CA). Horseradish peroxidase-conjugated anti-rabbit antibody and ECL^TM^ Western blotting system were obtained from GE Healthcare. The EIA commercial kit for plasma angiotensin I (Ang I) determinations was obtained from Bachem (Torrance, CA) and ^32^P_i_ was purchased from the Brazilian Institute of Energy and Nuclear Research (São Paulo, SP, Brazil). [γ-^32^P] ATP was synthesized as in [21]. The commercial creatinine kit was obtained from Labtest (Lagoa Santa, MG, Brazil). All other reagents were of the highest purity available.

### Animal groups

Seventy-day-old female Wistar rats, weighing 200–250 g, were randomly assigned to a maternal control group or saline group. Until weaning, the maternal control group (n = 4) had free access to tap water and the maternal saline group (n = 4) consumed 0.17 M NaCl (1% w/v). Salt loading *via* drinking water rather than food does not reflect the common situation in humans and does not allow a compensatory increase in free water consumption, and this could be considered as a limitation of the experimental model. In addition, it can evoke an augmented antidiuretic hormone (ADH) release and, consequently, in ADH plasma levels. However, we decided to overload mothers by giving salt in drinking water, aiming to program the fetuses without any maternal compensatory mechanism to correct disturbances in the circulating and tissular Na^+^. Maternal water intake was measured in a previous work with the same model of Na^+^ overload [2] and varies between the two dam groups. At pregnancy day 1 it was (in ml/24 h per 100 g body weight) 13.8±4.5 (control) and 27.3±2.4 (Na^+^-overloaded dams) (P<0.05); at day 18 it was 17.8±1.5 and 29.9±4.9 (P<0.05) in the respective groups.

Relating water ingestion to the amount of chow consumed (containing 0.3% NaCl), the daily maternal salt intake by Na^+^-overloaded dams averaged 700 mg. The salt intake in control dams averaged 100 mg per day. This ratio in salt intake regime cannot simply be extrapolated to the clinic. In the case of humans, a normal dietary NaCl averages 7%, whereas a content that varies between 10 and 20% (only two fold higher) is considered high as in some Eastern populations [22] and also in Northeastern Brazil [23]. In contrast, NaCl intake in rats is considered normal at 1.1% and elevated above 6% (*i.e.* six fold higher) as described in different models [4].

Mating was carried out at 90 days of age. Only male offspring was used for experimental protocol. The rats (dams and offspring after weaning) were provided with balanced commercial rodent chow (Purina Agribands, Paulínia, SP, Brazil). The control group (C, n = 19) comprised male offspring from mothers that consumed tap water throughout the study. Male offspring from dams that drank saline throughout the prenatal and lactation periods comprised the S group (n = 24). At birth, litters were culled to eight pups (including females that were used in other experiments after weaning) and maintained until weaning. After weaning (21 days after birth), 11 controls and 13 members of the S group were maintained with tap water. Others from each group were maintained on tap water supplemented with enalapril maleate (E; 100 mg/l) for three weeks, and with pure tap water thereafter; these subgroups were denoted by CE (n = 8) and SE (n = 11), respectively. Rats exposed to enalapril were administered an average of 4 mg per day during the three weeks after weaning. All experiments were carried out on animals aged 90 days.

We took into account the possibility of litter effects and, for this reason, pups from each litter are represented in each of the four experimental conditions. The 5–6 male offspring from each litter coming from the two groups of dams (control and Na^+^ overloaded) were randomly assigned to one of the two conditions to which they were submitted after weaning (without and with enalapril). Thus, we avoided litter effects and balanced the experimental design by using at least one rat from each litter (control mothers) or two rats from the different litters of Na^+^-overloaded dams.

### Blood pressure, creatinine clearance, proteinuria, urinary volume and urinary Na^+^ excretion

After acclimation of rats in the room and to the cage constraint for 30 min, systolic blood pressure (SBP) was measured from age of 25 days to 90 days in awake animals using tail-cuff plethysmography (IITC Life Science B60-7/16”, Life Science Instruments, Woodland Wills, CA). Acclimation of the animals was carried out along three days before pressure determinations. The room was preserved from noise, the ambient temperature was around 22°C and rats were warmed at 36±2°C. Metabolic cages (Tecniplast Gazzada Sarl, Buguggiate, Italy) were used to collect twenty-four h urine samples for measuring proteinuria, creatinine and Na^+^. Blood samples were obtained from the caudal artery for creatinine measurements. Urinary protein was measured using the Folin phenol method [24]. Urinary Na^+^ was measured using an electrolyte analyzer (AVL 9180, Roche Diagnostics GmbH, Mannheim, Germany). Serum creatinine was determined using a commercial kit. 48 h after completion of the metabolic studies, animals were decapitated and the kidneys removed to isolate the *cortex cortices* to obtain tubular plasma membranes (see below); the remaining tissue was used to evaluate thiobarbituric acid reactive substances (TBARS) as a measure of lipid peroxidation, constituting an estimate of renal oxidative stress.

### Evaluation of lipid peroxidation

Lipid peroxidation was assessed in kidney tissue by measuring TBARS according to Buege and Aust [25]. One gram tissue fragments were homogenized in 5 ml of 150 mM KCl in an ice bath. Two ml of 0.375% (w/v) thiobarbituric acid diluted with 15% TCA (w/v) were added to each ml of homogenate. The tubes were sealed and heated to 100°C for 15 min and centrifuged in a clinical centrifuge, and the absorbance of the resulting supernatants was evaluated at 535 nm.

### Isolation of proximal tubule cell membranes

Membranes were isolated as previously described [26] from the *cortex corticis*, a renal region where more than 90% of the cell population corresponds to proximal tubule cells [27], [28]. Kidneys were maintained in cold 250 mM sucrose, 10 mM HEPES-Tris (pH 7.4), 2 mM EDTA, 0.15 mg/ml trypsin inhibitor and 1 mM PMSF (solution A). Renal cortex was carefully dissected to eliminate contamination with internal regions of the organ and thin transverse sections (0.5 mm) were cut using a Stadie-Riggs microtome. The fragments were homogenized in 4 ml of solution A per gram of tissue in an ice bath. The homogenate was centrifuged at 755× *g* (15 min) to remove unbroken cells, cell debris and nuclei; the resulting supernatant was centrifuged at 8,500× *g* (20 min) to remove mitochondria and at 35,000× *g* (45 min). The final sediment was resuspended in 250 mM sucrose, aliquoted into tubes and stored at −20°C. Protein content was determined using the Folin phenol method [24] with BSA as a standard, using 2.5% (w/v) SDS to solubilize integral membrane proteins. Control for enrichment with basolateral membranes (3−4 fold with respect to the total homogenate) and for minimal residual contamination with other intracellular membranes were as described elsewhere [26]. The final fraction contained apical membranes at a lower yield than the starting homogenate, as revealed using alkaline phosphatase assays [26]. However, ATP-driven Na^+^ transporters are exclusively located in the basolateral aspect of the cell membrane. Therefore, there was no attempt to fractionate the samples further. The Percoll gradient method used to separate brush border and basolateral membranes from porcine and ovine kidneys [29], [30] results in a very low yield of membranes from rat kidneys. Therefore, avoiding this step allowed a reduced number of rats to be utilized during this study, as recommended by the local Committee for Ethics in Animal Experimentation.

### Measurement of ATPase activities

The activity of ouabain-sensitive (Na^+^+K^+^)ATPase was measured colorimetrically using unlabeled ATP. During (Na^+^+K^+^)ATPase assays, membranes (0.1 mg/ml final concentration) were pre-incubated with or without 2 mM ouabain in 0.1 ml water at 37°C for 20 min. The assays were supplemented with 50 mM Bis-Tris-propane (pH 7.4), 0.2 mM EDTA, 5 mM MgCl_2_ and 120 mM NaCl (final concentrations in 0.5 ml assays). The reaction was started by adding a mixture of 5 mM ATP and 24 mM KCl (final concentrations), and stopped after 10 min by adding two vol of 0.1 M HCl-activated charcoal. The (Na^+^+K^+^)ATPase activity was calculated as the difference between P_i_ released in the absence and presence of ouabain. Released P_i_ was spectrophotometrically measured in an ml aliquot of the supernatant obtained after centrifugation of the charcoal suspension at 1,500× *g* for 5 min.

The ouabain-resistant, furosemide-sensitive Na^+^-ATPase activity was measured using [γ-^32^P] ATP (∼0.03 MBq/µmol) or unlabelled ATP; each method produced identical results (P>0.05) and the measurements were grouped for final statistical analysis. The activity was calculated as the difference between P_i_ or ^32^P_i_ released in the absence and presence of 2 mM furosemide. The reaction was started by adding 5 mM ATP (or [γ-^32^P] ATP) to the membranes (0.2 mg/ml) pre-incubated with 2 mM ouabain, as described above, in the presence of 20 mM HEPES-Tris (pH 7.0), 10 mM MgCl_2_ and 120 mM NaCl. After 10 min the reaction was stopped. Released ^32^P_i_ was measured using liquid scintillation counting or spectrophotometrically in (P_i_) a 0.2 ml aliquot of the supernatants obtained.

In one series of experiments, Na^+^-ATPase activity was evaluated in basal conditions (no Ang II) and, in other three and simultaneous series of tubes, in the presence of different concentrations of Ang II (10^−12^, 10^−10^ and 10^−8^ M). The impact of PKA inhibition on the activity of the pump was evaluated using the specific inhibitor PKAi_5–24_ (PKAi) during Na^+^-ATPase assays.

### Protein kinase C (PKC) and cAMP-dependent protein kinase (PKA)

Protein kinase activities were analyzed by measuring the incorporation of the γ-phosphoryl group of [γ-^32^P] ATP (specific activity ∼1 MBq/nmol) into histone in the absence and presence of the specific PKC or PKA inhibitors, 100 nM calphostin C and 10 nM PKAi, as described previously [29]. The reaction was started by adding ATP (10 μM) to a reaction medium (0.1 ml) containing 20 mM HEPES-Tris (pH 7.0), 4 mM MgCl_2_, 1.5 mg/ml histone and 0.7 mg/ml tubule membranes protein. After 2 min, the reaction was stopped by adding 0.1 ml 40% TCA and the samples were immediately placed on ice bath. After vigorous stirring, a 0.1 ml aliquot was removed, filtered through a Millipore filter (0.45 μm pore size) and successively washed with ice-cold 20% TCA and 0.1 M phosphate buffer (pH 7.0). Radioactivity was quantified using a liquid scintillation counter. Protein kinase activities were calculated as the difference between total ^32^P incorporated into histone and incorporation in the presence of calphostin (for PKC) or PKAi (for PKA).

### Immunoprecipitation of Ang II receptors (AT_1_ and AT_2_ receptors)

Immunoprecipitation was carried out before immunodetection of AT_1_ and AT_2_ receptors, as they are expressed at very low levels in proximal tubule membranes [30]. Isolated membranes (1 mg/ml) were initially solubilized in a sucrose solution containing 0.01% CHAPS for 30 min at room temperature. Primary polyclonal antibodies (1∶400 dilution) were mixed with protein A-agarose, gently stirred for 20 min and supplemented with an equal volume of BSA (1 mg/ml) in 0.01% (w/v) CHAPS. This mixture was added to the isolated membrane samples. After constant stirring overnight at 4°C, the samples were centrifuged at 1,000× *g* for 4 min. The supernatant was retained as a control for the immunoprecipitation procedure. The pellet was washed three times with Tris-buffered saline (TBS) and heated to 100°C in a water bath for 4 min with 40 µl SDS-PAGE sample buffer. After final centrifugation at 10,000× *g* for 2 min, the supernatant was subjected to SDS-PAGE and Western blotting.

### SDS-PAGE and Western blotting

The α-subunit of (Na^+^+K^+^)ATPase and the Ang II receptors were detected in the membranes and immunoprecipitates, respectively, using specific antibodies. Sample proteins were separated using SDS-PAGE (10%) and transferred to nitrocellulose membranes at 350 mA. Non-specific binding was prevented by incubating the membranes with 5% non-fat milk diluted in TBS (pH 7.6) for 1 h. The membranes were probed with the corresponding primary antibodies (1∶500 dilution) for 1 h at room temperature during gentle stirring, washed three times with TBS containing 0.1% Tween 20, exposed to the secondary antibody, washed and visualized using ECL™.

### Immunohistochemical and morphometrical analysis

Transverse slices of kidneys (3 mm) were fixed in Methacarn for 24 h and maintained in 70% ethanol until encapsulated in paraffin. Sections of 6 µm were used for morphometrical and immunohistochemical analysis of cortical and medullary regions. For morphomometrical studies and evaluation of collagen density, sections were stained with periodic acid-Schiff and Sirius red, respectively. For immunohistochemical evaluation of macrophage infiltration, sections were incubated overnight with a monoclonal antibody anti-ED1 (1∶100) in a humid chamber at 4°C, and the reaction product was visualized using a secondary antibody labeled with streptavidin-horseradish peroxidase under a light microscope (Eclipse 400, Nikon, Shanghai, China) coupled to a camera (Evolution, Media Cybernetics Inc., Bethesda, MD). For ED1 and collagen evaluation, 20 images from the medulla and cortex of each kidney were acquired using ×400 and ×200 magnification, respectively. The surface density of collagen and ED1 was identified by a researcher and counted using the Image Pro Plus 4.5.1 software (Media Cybernetics, Bethesda, MD). Immunostaining for Ang II in renal cortical cells was carried out as previously described [31] in slices prepared and incubated with the corresponding antibody (1∶200). The results in the graphic representations of collagen and ED1 correspond to the ratio between positive areas and the total area under observation. Ang II-positive cells in the tubulointerstitial region were counted in 60 fields from various kidneys (×100 magnification). Measurement of Ang II-positive cells was carried out using 60 glomeruli randomly chosen from kidneys from different rats (×200 magnification). Similarity among slides from the same rat was used as a criterion of unbiased data. Morphometrical studies concerning glomeruli were performed with 30 images being acquired using ×200 magnification and analyzed using AxioVision software (version 4.8.1.0, Carl Zeiss Imaging Solutions).

### Determination of Ang I levels in plasma

Blood samples (4 ml) obtained after animal decapitation were collected in tubes supplemented with concentrated solutions of EDTA (disodium salt), PMSF and trypsin inhibitor, producing final concentrations of 2.7 mM, 1 mM and 0.15 mg/ml, respectively, in a final volume of 5 ml. After centrifugation at 1,600× *g* the supernatants were frozen at −70°C until determinations were carried out. After thawing, samples were submitted to ELISA following the manufacturer's instructions (protocol III) and the final results were expressed as ng per ml of the original plasma sample.

### Statistical analysis

Differences among groups were analyzed using one-way ANOVA test followed by a Student-Newman-Keuls post test, or two-way ANOVA followed by Bonferroni post test, when corresponding. One-way ANOVA followed by Tukey's test analysis was used to compare the responses of Na^+^-ATPase to Ang II within groups. Kruskal-Wallis test followed by Dunn's multiple comparison test was used to analyze creatinine clearance, proteinuria and TBARS data, after detection of their departure from normality using the Kolmogorov-Smirnov, D'Agostino & Pearson and Shapiro-Wilk normality tests. GraphPad Prism 5 software (Version 5.01, GraphPad Software, Inc. La Jolla, CA) was used for all statistical analyses. The statistical differences were considered significant at P<0.05.

## Results

### Body weight at birth and weaning

The birth body weight of offspring was not affected by maternal saline treatment (C: 6.41±0.18 and S: 6.17±0.19 g), as well as the weaning body weight (C: 49±2 and S: 48±3 g).

### Evolution of systolic blood pressure (SBP) with age

**Figure 1:**
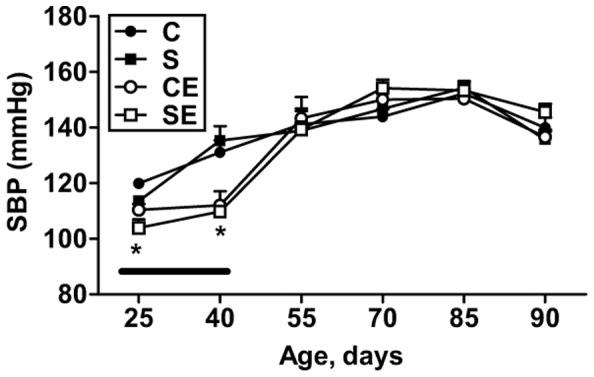
Systolic blood pressure (SBP) during growth, from age of 25 days to 90 days. SBP was measured at the ages shown on the *abscissa*. The symbols relating to the groups C, S, CE and SE are indicated in the inset. The continuous horizontal thin black bar indicates the period of daily administration of enalapril to the CE and SE groups. Results are means ± S.E.M. * Statistical difference (P<0.05) between CE and SE with respect to C and S.

SBP from 25−90 day old rats is presented in Fig. 1. Perinatal Na^+^ overload does not lead to the onset of hypertension in offspring during the period under study. Three-week daily administration of enalapril from weaning provoked a significant decrease in SBP (compare C and S with CE and SE during the period indicated by the horizontal line), demonstrating that the ACE was impacted by the drug. A complete recovery of control values occurred after treatment was ceased. Furthermore, at 90 days of age, SBP of each group increased by approximately 12% after exposure to ammonia (data not shown) to induce olfactory stress [32]. We tested the effect of ammonia on blood pressure to see whether or not the normalized blood pressure in the programmed offspring responds in the same extent as the other groups to the pressoric olfactory stress, as it was the case.

### Water balance and excretion of Na^+^ by the offspring

Table 1 depicts results regarding water intake, diuresis and 24 h-urinary Na^+^ excretion. Alterations in Na^+^ excretion were observed at 30 days, likely reflecting maternal Na^+^ overload in the S group and an early and exacerbated response to enalapril in the CE group. Urinary Na^+^ excretion was similar in all groups at 90 days of age. Water intake and diuresis were similar in all groups and decreased in parallel with age, probably reflecting the elevated water demand at early stages of life.

**Table 1:** Water balance and excretion of Na^+^ by the offspring 1.

	Day	C	S	CE	SE
Offspring water intake 2	30	30.5±4.1	34.0±1.6	29.5±1.5	36.3±2.3
	90	10.2±0.4	11.4±0.6	11.3±0.3	11.9±0.5
Offspring water excretion 3	30	11.6±2.4	7.3±0.9	10.7±0.9	9.3±0.9
	90	3.6±0.2	3.5±0.1	3.8±0.2	3.6±0.2
Offspring urinary Na^+^ 4	30	0.47±0.02	0.58±0.04 *	0.72±0.03 *^,†^	0.62±0.03
	90	0.57±0.03	0.56±0.02	0.56±0.02	0.54±0.02

1 n = 7–8; 30 and 90 indicate postnatal day of life.

2 In ml/24 h per 100 g BW.

3 In ml/24 h per 100 g BW; * P<0.05 with respect to C.

4 In ml/24 h per 100 g BW; * P<0.05 with respect to C; ^†^ P<0.05 with respect to S.

### (Na^+^+K^+^)ATPase and Na^+^-ATPase

**Figure 2:**
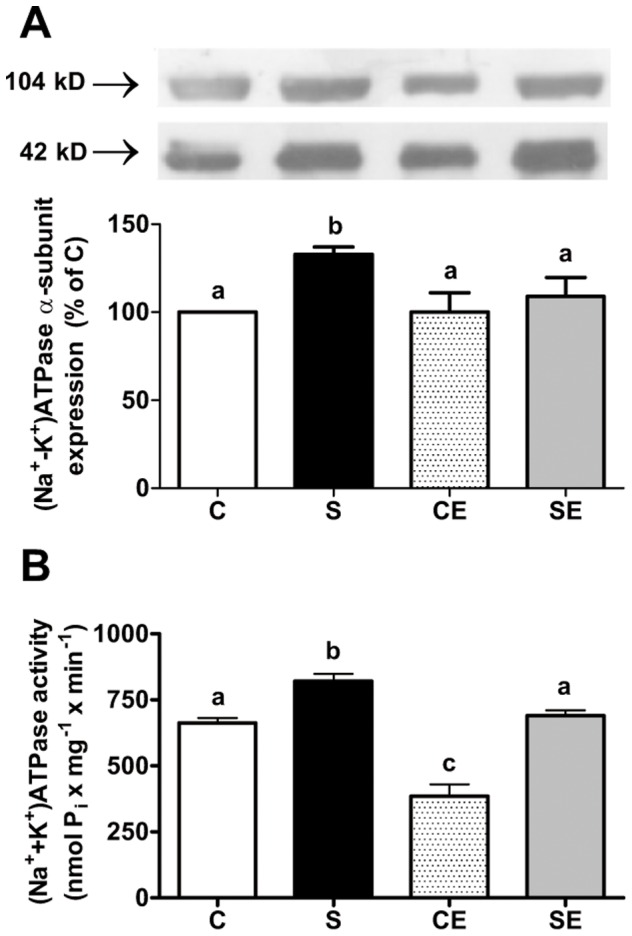
Expression and activity of (Na^+^+K^+^)ATPase in membranes of proximal tubule cells. (A) upper and middle panels demonstrate representative immunoblots of (Na^+^+K^+^)ATPase α-subunit (104 kD) and β-actin (42 kD), respectively. Lower panel: densitometric representation of α-subunit expression corrected by corresponding β-actin immunostaining (same lane); n = 8 (C and S) and n = 9 (CE and SE). Densitometric records were converted to percent values taken as 100% that of the C group obtained from the same nitrocellulose membrane. B: (Na^+^+K^+^)ATPase activity. Results are means ± S.E.M.; n = 6 (C and S), n = 5 (CE) and n = 7 (SE). Different lowercase letters above the bars indicate statistical difference (P<0.05).

Fig. 2A demonstrates that programming caused by perinatal Na^+^ overload increased expression of the α-subunit of (Na^+^+K^+^)ATPase in basolateral membranes of proximal tubules from 90 day old offspring (S). Treatment with enalapril for three weeks after weaning resulted in expression of the pump to return to control values (SE), and there was no effect in non-programmed animals (CE). When enzyme activity was measured (Fig. 2B), perinatal Na^+^ overload programmed elevated activity of (Na^+^+K^+^)ATPase (S), and treatment with enalapril reprogrammed the enzyme (SE), resulting in activity at levels exhibited by the C group. In contrast, enalapril treatment lowered (Na^+^+K^+^)ATPase activity by approximately 50% in C animals.

**Figure 3:**
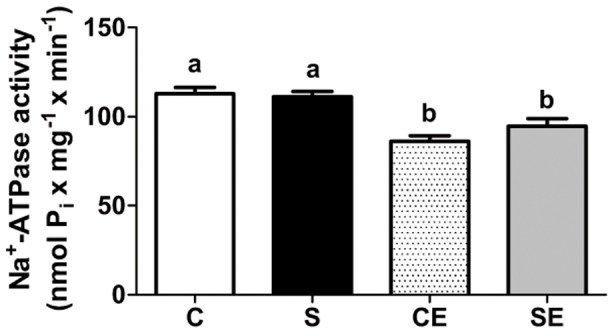
Activity of the ouabain-insensitive Na^+^-ATPase in membranes of proximal tubule cells. Values are means ± S.E.M.; n = 26 (C), n = 23 (S), n = 22 (CE) and n = 21 (SE). Different lowercase letters above the bars indicate statistical difference (P<0.05).

**Figure 4:**
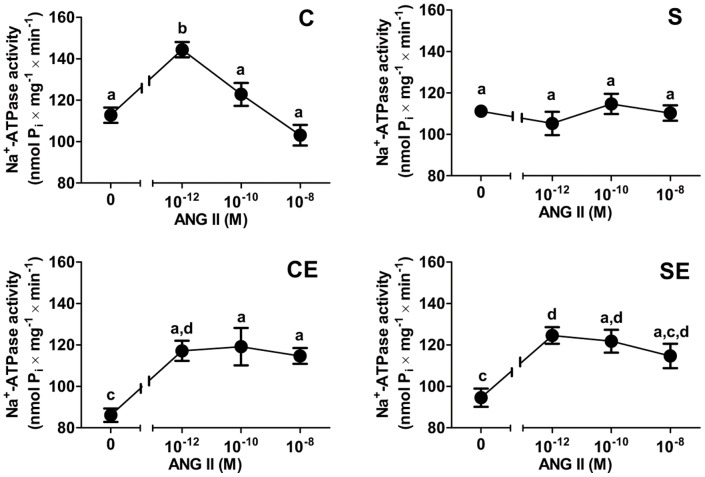
Responsiveness of ouabain-insensitive Na^+^-ATPase activity to Ang II. Panels C (upper left) and S (upper right) correspond to the offspring from control and perinatally Na^+^-overloaded mothers, respectively. Panels CE (lower left) and SE (lower right) correspond to the previous two groups treated with enalapril. Values are means ± S.E.M.; (n = 5–26 depending on the group and Ang II concentrations shown on the *abscissae*). Different lowercase letters above the circles indicate differences (P<0.05) calculated by comparing mean values within and among figures. Two or three letters above the same bar (a, d; a, c, d) relate to the fact that the corresponding mean value is not different from the others.

The activity of ouabain-insensitive Na^+^-ATPase (Fig. 3) was not affected by perinatal Na^+^ overload (C and S). However, enalapril reduced ouabain-insensitive Na^+^-ATPase activity in control rats and the progeny of Na^+^-overloaded mothers (CE and SE) by comparable amounts. Na^+^-ATPase activity was measured in the presence of different Ang II concentrations (Fig. 4). The effect of the peptide was biphasic in the C group: 10^−12^ M Ang II significantly increased Na^+^-ATPase activity and high concentrations led to progressive inhibition of the previously stimulated activity (upper left panel); without exogenous Ang II, Na^+^-ATPase activity remained at levels observed in the C group (Fig. 3). The effect of Ang II was lost in the S group at each concentration assayed (upper right panel). In the non-programmed group treated with enalapril (CE, lower left panel), 30%-stimulation using 10^−12^ M Ang II was preserved – albeit at lower absolute values – with loss of the biphasic profile. In the SE group (lower right panel), percentual stimulation elicited by 10^−12^ M Ang II was maintained at lowered absolute values without significant inhibition at higher concentrations.

### AT_1_ and AT_2_ receptors density

**Figure 5:**
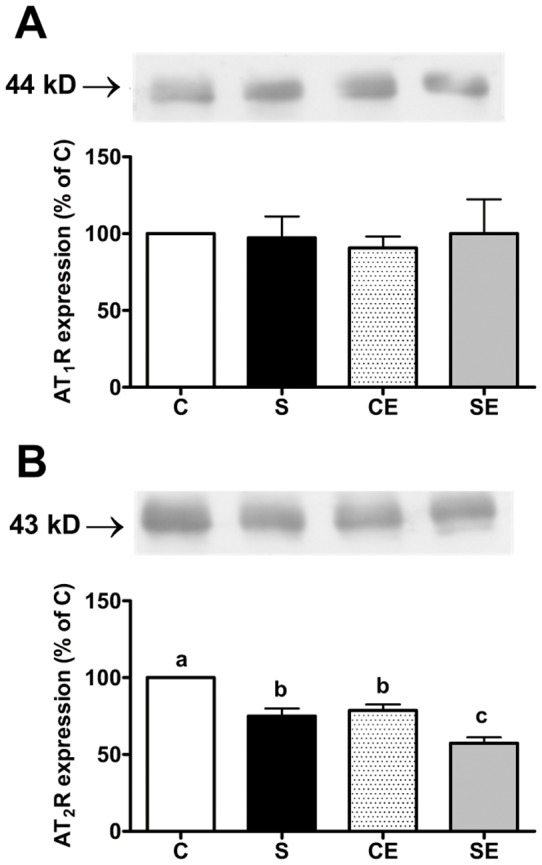
AT_1_ receptors and AT_2_ receptors expression in membranes of the proximal tubule cells. AT_1_ (A) and AT_2_ (B) receptores. Upper panels: representative immunodetections. Lower panels: densitometric representations of receptor immunosignals corrected for protein loading (ponceau red) after immunoprecipitation. Values are means ± S.E.M.; (n = 5 for AT_1_; n = 3–5 for AT_2_). C values were taken as 100% and those from S, CE and SE groups in the same gel were expressed as a percentage of C (n = 3–5 for AT_2_; n = 5 for AT_1_). Different lowercase letters above bars indicate statistical differences (P<0.05).

To investigate whether Na^+^- and enalapril-induced changes in Na^+^-dependent ATPases activities (Fig. 2 and Fig. 3) and kinase activities (see below) were due to alterations at the beginning of the RAS cascade, the expression of AT_1_ and AT_2_ receptors in proximal tubule membranes were analyzed in 90 day old rats. Maternal Na^+^ overloading and enalapril treatment after weaning had no effect (alone or combined) on AT_1_ receptor expression (Fig. 5A). However, perinatal Na^+^ overload programmed down-regulation of AT_2_ receptors in adult offspring (S), an effect that was provoked to a comparable extent by enalapril treatment in non-programmed animals (CE). The more accentuated down-regulation observed in the SE group appeared to indicate that the effects of each treatment are additive.

### PKC and PKA activities and impact of specific inhibition of PKA

**Figure 6:**
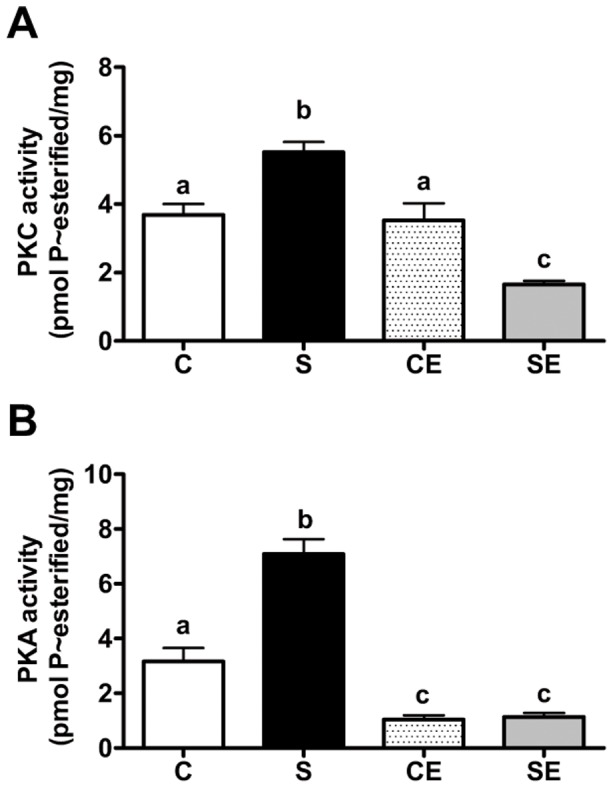
PKC activity and PKA activity in proximal tubule cell membranes. PKC activity (A) and PKA activity (B). Values are means ± S.E.M. (n = 3 for PKC and n = 4–5 for PKA). Different lowercase letters above bars indicate statistically different values (P<0.05).

Fig. 6A demonstrates that PKC activity in renal membranes was increased by 50% in the S group compared with the C group. Enalapril treatment after weaning did not affect PKC activity in control rats (CE) but promoted a strong decrease in prenatally Na^+^ overloaded rats (SE). PKA was increased by more than 100% in S rats (Fig. 6B), and treatment with enalapril reduced PKA activity in programmed and non-programmed groups to comparable very low levels (CE and SE).

**Figure 7:**
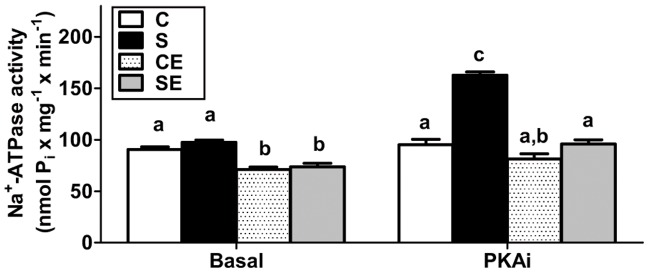
Ouabain-insensitive Na^+^-ATPase activity measured in the absence or presence of PKAi_5-24_ peptide. Absence (basal condition, left) or presence (right) of PKAi_5–24_ peptide. The results depicted in Fig. 3 (basal) are presented and statistically analyzed in conjunction with those obtained in the presence of PKAi (n = 7). Results are means ± S.E.M. Different lowercase letters above bars indicate statistical differences (P<0.05). Two letters above the same bar (a, b) relate to the fact that the corresponding mean value is not different from others.

Fig. 7 presents data regarding the influence of inhibition of PKA on Na^+^-ATPase activity in the various offspring groups. When the assays were supplemented with PKAi to shut down PKA, the most remarkable effect was a greater than 65% increase in the activity of the pump in the S group.

### Renal TBARS, creatinine clearance and proteinuria

**Figure 8:**
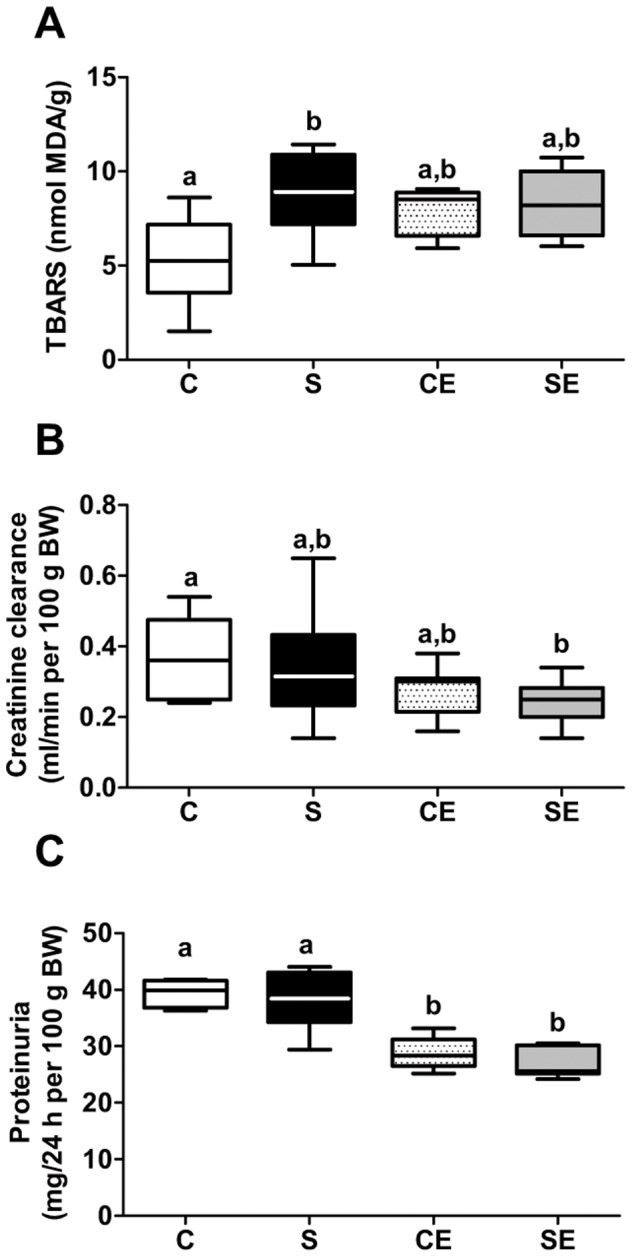
Thiobarbituric acid reactive substances (TBARS) in renal tissue, creatinine clearance and proteinuria. TBARS (A), creatinine clearance (B) and proteinuria (C). The box plots in the three panels present the median, minimum and maximum values; n = 8–12 (A), n = 13–16 (B) and n = 7–8 (C). Different lowercase letters above bars indicate statistical differences (P<0.05). Two letters above the same bar (a, b) relate to the fact that the corresponding mean value is not different from others.

Fig. 8A demonstrates that perinatal Na^+^ overloading markedly increased renal TBARS in the S group. Administering enalapril to non-programmed rats (CE group) increased TBARS, but did not augment cumulatively the TBARS levels in programmed rats (SE group). With respect to creatinine clearance (Fig. 8B), there was no modification in S rats, and the most striking effect – a significant decrease – was observed in the SE group, with a less pronounced effect in CE animals. Enalapril decreased proteinuria regardless of whether rats were exposed to high levels of Na^+^ during prenatal and lactational periods.

### Immunohistochemical and morphometrical data

**Figure 9:**
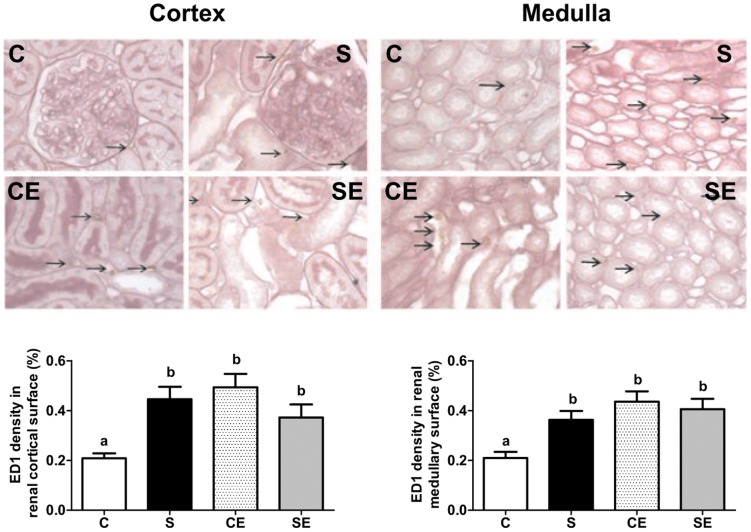
Macrophage infiltration of cortical and medullary regions evaluated by immunostaining of ED1 antigen. Upper and middle panels are representative fields (×400) of cortex and medulla from C, S, CE and SE experimental groups. Arrows point to ED1 positive cells. Lower panels present percentage values of ED1 surface density per field in cortex (left) and medulla (right). Different lowercase letters above bars indicate statistically different values (P<0.05).

**Figure 10:**
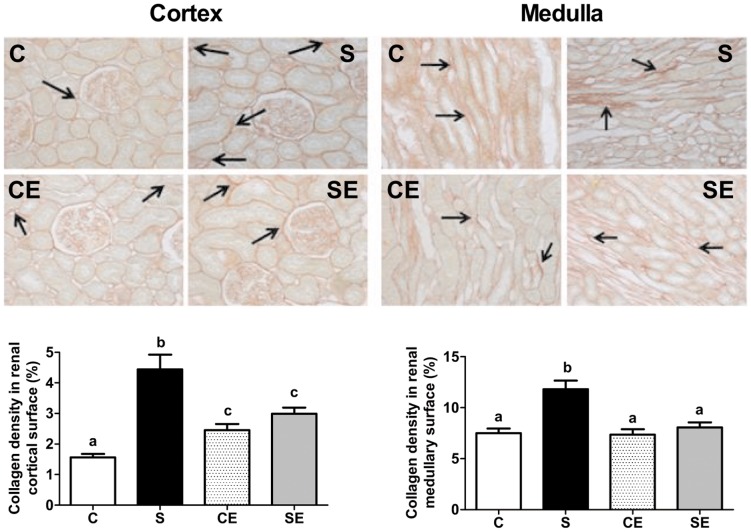
Collagen staining of cortical and medullary regions. Upper and middle panels are representative fields (×200) of cortex and medulla from the C, S, CE and SE experimental groups. Arrows point to collagen deposition. Lower panels present percentage values of collagen per field in cortex (left) and medulla (right). Different lowercase letters above bars indicate statistically different values (P<0.05).

Fig. 9 demonstrates that macrophage infiltration increased in the renal cortical and medullary regions in the S, CE and SE groups. Collagen density (Fig. 10) increased by more than 150% in the cortex and by 60% in the medulla of rats in the S group, an effect that was reversed to a great extent (cortex) or completely (medulla) by enalapril administration. Enalapril alone increased collagen deposition (CE) but to a lower extent than that observed with cortical ED1 antigen (compare left graph bars in Figs. 10 and 9).

Morphometrical analysis demonstrated that the areas of glomeruli, glomerular capillary tufts and urinary spaces within glomeruli, remained unaltered independent of the treatment administered (data not shown).

### Plasma levels of Ang I

**Figure 11:**
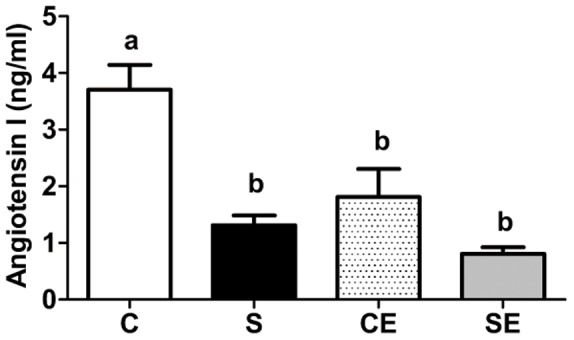
Plasma levels of Ang I. Values are means ± S.E.M. of 6–8 determinations using different rats, carried out in duplicate. Different lowercase letters above bars indicate statistical differences in mean values (P<0.05).

Plasma levels of Ang I were measured to investigate whether perinatal Na^+^ overload programs alterations in the systemic RAS of the adult offspring. The data depicted in Fig. 11 demonstrate that perinatal Na^+^ overloading programmed a decrease of more than 60% in circulating Ang I in adult offspring (S). In enalapril-treated rats (CE and SE), Ang I levels were comparable to those in the S group.

**Figure 12:**
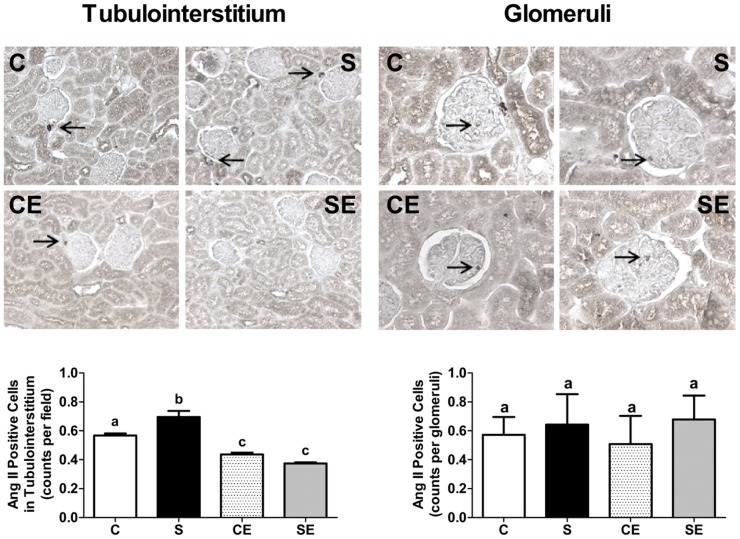
Immunodetection of Ang II in the renal cortex. Upper and middle panels are representative fields demonstrating Ang II positive cells in the C, S, CE and SE groups counted in tubulointerstitium (×100) and glomeruli (×200), as indicated at the top of the figure. Arrows point to Ang II positive cells. Lower panels present graphic representations of counting (means ± SE). Different lowercase letters above bars indicate statistical differences (P<0.05). Each mean (n = 5) resulted from counting 60 fields in each kidney.

### Ang II determinations in renal cortex

The number of Ang II-positive cells in the tubulointerstitial region increased by 25% with respect to controls as a result of perinatal Na^+^ overload (Fig. 12, compare C and S), and enalapril treatment caused a comparable decrease in the CE and SE groups. When the number of Ang II-positive cells was counted in glomeruli, the number was the same regardless of whether the animals were submitted to perinatal Na^+^ overload or not, or to enalapril treatment after weaning (Fig. 12).

## Discussion

The present study describes results that partially elucidate renal molecular mechanisms underlying alterations in active Na^+^ transport across proximal tubule epithelium and local Ang II pathways in young adult offspring, which were programmed due to maternal Na^+^ overload during gestation and lactation. Furthermore, this research demonstrates that short-term treatment with enalapril after weaning can reprogram much of the Na^+^ overload-induced renal alterations in progeny, but paradoxically, in some cases this treatment can mimic or accentuate the impact of Na^+^ overload.

Permanent up-regulation of the Na^+^ pump responsible for the majority of Na^+^ reabsorption in proximal tubules, (Na^+^+K^+^)ATPase [19], is evident in Fig. 2. This may be the result of erroneous adaptive signaling processes involving nuclear and cytosolic factors and enzymes such as PKC [33], [34], activated as a consequence of low level apical Na^+^ entry. This incorrect signal could be elicited by the retraction of brush border Na^+^ transporters, viewed as a response to increased luminal Na^+^ during Na^+^ overloading and augmented filtered Na^+^ [35]. Up-regulated PKC (Fig. 6A) could be responsible for these alterations by direct regulatory phosphorylation of the α-catalytic subunit, impacting enzyme turnover [36]. In addition, programmed up-regulation of PKA (Fig. 6B), which stimulates (Na^+^+K^+^)ATPase in several tissues [37], [38], may be another incorrect signal that accounts for direct and permanent stimulation of (Na^+^+K^+^)ATPase above normal levels. With respect to expression of (Na^+^+K^+^)ATPase, PKA promotes targeting of pump units in basolateral membranes of proximal tubules [38], [39], which was evident in the adult offspring during this study (Fig. 2A). However, despite this plausible explanation, an opposite mechanism could be responsible for the up-regulation of (Na^+^+K^+^)ATPase: the stimulus resulting from a long term increased apical Na^+^ entry. This alternative view is supported by the observation that high Na^+^ intake increases removal of fluid from the lumen of rat proximal tubules [40], an indication of augmented primary Na^+^ flux across the apical membrane.

The ouabain-resistant, furosemide-sensitive Na^+^-ATPase has been cloned and purified recently [41] and, in kidney, it is considered responsible for fine tuning of Na^+^ reabsorption in proximal tubules [20], [42]. Na^+^-ATPase, which is modulated *in vitro* by hormones and autacoids that participate in the physiological regulation of extracellular fluid [42], [43], was unaltered in the membranes of animals in the S group (Fig. 3). A constitutive increase in activity was expected facing the up-regulation of PKC (Fig. 6A), which plays a role in its physiological activation [20]. However, the significant counteracting increase in PKA activity could negate the influence of PKC [43], [44] in rats programmed by Na^+^ overload. This is an idea that receives support from the observation that the PKA inhibitor peptide elicited a pronounced elevation of Na^+^-ATPase (Fig. 7). Therefore, the effect of PKA action on Na^+^-ATPase was different from our previous results [44] and those from other laboratories [42]. However, the stimulatory effect of physiological Ang II concentrations on Na^+^-ATPase correlates with PKC [20], [43], and the loss of the normal biphasic influence of the peptide (Fig. 4, upper right panel) could be due to the imbalance between PKC and PKA. It is of special interest in the context of the present work that a recent phosphoproteomic-based study demonstrated that several effector proteins can be selectively phosphorylated in response to Ang II [45].

The loss of the inhibitory effect of high Ang II concentrations in animals from the S group (Fig. 4) could be due to a programmed decrease in the expression of AT_2_ receptors (Fig. 7A), the first step in the Ang II-associated cascade that culminates in the inhibition of Na^+^-ATPase caused by high concentrations of the peptide [46]. Despite the lower expression of AT_2_R in kidney, there is growing evidence about their crucial role in renal physiological and pathological processes. For example, beneficial effects on renal injury achieved with AT_1_R blockade are potentiated by Ang II effects transduced through AT_2_R, in a model where collagen deposition is significant [47], as in our model (Fig. 10).

Perinatally Na^+^ overloaded adult offspring presented with increased macrophage infiltration in the whole kidney, and collagen deposition in the cortex that was not counteracted by enalapril treatment (Figs. 9 and 10). However, it is striking that enalapril *per se* augmented the inflammatory response, and promoted modest but significant collagen deposition, macrophage infiltration and TBARS increment, as ACE inhibition ameliorates several pathological indicators including local oxidative stress in uremic rats [48]. Decreased proteinuria (Fig. 8C) was in accordance with the known beneficial actions of RAS blockade. Despite the experimental contradiction, it is possible that a common factor, increased ROS production, is involved in these structural alterations programmed in perinatally Na^+^ overloaded rats. High oxidant production has been correlated with renal inflammation indicators [7], [49], [50], and consequently it is plausible that ROS contributed to macrophage infiltration and collagen deposition as a legacy imprinted by enalapril administration in the short window of 21 days after weaning. Regarding the latter process, the significant increase in Ang II levels in the cortical tubulointerstitium of the S group (Fig. 12) could be another key factor that contributed to collagen deposition in association with ROS. Recently, it was demonstrated that Ang II-induced ROS play a central role in the signaling cascade that accentuates collagen deposition in renal tissue, which can culminate in fibrosis [51].

At this point a question emerges. How would enalapril induce increased ROS production? It is likely that the drug impacted two physiological processes, oxidant production (Fig. 8A) and the glomerular filtration rate (GFR), at the same time. Its considerable impact on creatinine clearance (Fig. 8B), through a common mechanism of reducing blood pressure during administration after weaning (Fig. 1, empty points above the horizontal line) has been demonstrated. Reduced blood pressure is likely to have led to lower renal blood flow and renal hypoxia. Programming vasodilatation of the efferent arteriole as a consequence of local RAS inhibition and decreased local Ang II (Fig. 12), could explain the reduction of GFR during adult life (Fig. 8B).

Interestingly, enalapril provoked a decrease in AT_2_ receptor expression (Fig. 5B), a side effect that reinforces the idea of simultaneous actions of the drug beyond its action in reprogramming (Na^+^+K^+^)ATPase expression and turnover (Fig. 2). The additive effects of perinatal Na^+^ overload and enalapril treatment on AT_2_ receptor expression (SE, Fig. 5B) clearly demonstrates there are separate pathways in which RAS participate, affecting renal molecular programming and reprogramming. The effects of enalapril treatment after weaning support the view that renal programming continues after nephrogenesis is complete [17]. The influences of Na^+^ overload and enalapril treatment are not additive in terms of effects on renal TBARS (Fig. 8A). Therefore, they are likely to impact on a common final enzymatic target in tubule cells, thereby impairing ROS detoxification.

Perinatal Na^+^ overload programmed two opposite alterations regarding RAS during adulthood. First, it strongly depressed levels of circulating Ang I, probably as a result of inhibiting systemic renin release that persisted in adult life. Indeed, plasma renin activity is lowered during high Na^+^ overload [52]. Second, high perinatal Na^+^ programmed elevated enalapril-sensitive levels of Ang II in cortical tubulointerstitium. The programming of enalapril-sensitive increased levels of local Ang II caused by Na^+^ overload, impacted (Na^+^+K^+^)ATPase and the ouabain-resistant Na^+^-ATPase as demonstrated in Figs. 2, 3 and 4, thereby contributing to increased Na^+^ reabsorption across proximal tubular epithelium, as is the case during continuous infusion of Ang II [53].

However, perinatally Na^+^-overloaded rats did not present with modifications in the levels of local Ang II in glomeruli (Fig. 12), and this correlates with a lack of alterations in the glomerular capillary tufts and the Bowman's capsule (data not shown). These negative results are important, as they reinforce the idea that local variations in Ang II levels and associated signaling pathways can be ascribed to structural and functional alterations in a renal tissue impacted by Na^+^ overload programming.

Finally, despite the important structural and functional renal alterations induced by perinatal Na^+^ overload, the rats were not hypertensive at 90 days old. Lower levels of circulating Ang I (Fig. 11) in the S group − as the result of an impact in systemic RAS − could be responsible for these low pressure levels. Salt loading via drinking water, as used in the present study, differs from high salt food intake by humans in that there was no scope for compensatory water intake and so, this method of salt loading may be a limitation. Nevertheless, it is clear that the ensemble of programmed alterations described herein could be the basis for the expansion of extracellular compartments, the increase in bodily Na^+^ content [22] and, possibly, the onset and maintenance of hypertension in older rats. Longer term experiments are now carried out to test this hypothesis. However, early treatment with enalapril reprogrammed and reduced the majority of alterations with regards to Na^+^ reabsorption and local Ang II levels, but side effects, particularly those associated with increased lipid peroxidation, down-regulation of AT_2_ receptors, and significant modifications to kinase activities, GFR, macrophage infiltration and cortical collagen deposition, could have an overall negative impact on renal function in older rats.
